# A Simple Method for the Design and Development of Flavivirus NS1 Recombinant Proteins Using an In Silico Approach

**DOI:** 10.1155/2020/3865707

**Published:** 2020-02-13

**Authors:** Chulmin Park, Won-Bok Kim, Sung-Yeon Cho, Eun-Jee Oh, Hyeyoung Lee, Kyungjoon Kang, Yoonsuk Lee, Dong-Gun Lee

**Affiliations:** ^1^Vaccine Bio Research Institute, College of Medicine, The Catholic University of Korea, Seoul 06591, Republic of Korea; ^2^Division of Infectious Diseases, Department of Internal Medicine, College of Medicine, The Catholic University of Korea, Seoul 06591, Republic of Korea; ^3^Department of Laboratory Medicine, Seoul St. Mary's Hospital, College of Medicine, The Catholic University of Korea, Seoul, Republic of Korea; ^4^Department of Laboratory Medicine, International St. Mary's Hospital, College of Medicine, Catholic Kwandong University, Incheon 22711, Republic of Korea; ^5^Boditech Med Inc., 43 Geodudanji 1-gil, Dongnae-myeon, Chuncheon, Gangwon-do 24398, Republic of Korea

## Abstract

Even in countries that are currently not facing a flavivirus epidemic, the spread of mosquito-borne flaviviruses presents an increasing public threat, owing to climate change, international travel, and other factors. Many of these countries lack the resources (viral strains, clinical specimens, etc.) needed for the research that could help cope with the threat imposed by flaviviruses, and therefore, an alternative approach is needed. Using an in silico approach to global databases, we aimed to design and develop flavivirus NS1 recombinant proteins with due consideration towards antigenic variation. NS1 genes analyzed in this study included a total of 6,823 sequences, from Dengue virus (DENV), Japanese encephalitis virus (JEV), West Nile virus (WNV), Zika virus (ZIKV), and Yellow fever virus (YKV). We extracted and analyzed 316 DENV NS1 sequence types (STs), 59 JEV STs, 75 WNV STs, 30 YFV STs, and 43 ZIKV STs using a simple algorithm based on phylogenetic analysis. STs were reclassified according to the variation of the major epitope by MHC II binding. 78 DENV epitope type (EpT), 29 JEV EpTs, 29 WNV EpTs, 12 YFV EpTs, and 5 ZIKV EpTs were extracted according to their major epitopes. Also, frequency results showed that there were dominant EpTs in all flavivirus. Fifteen STs were selected and purified for the expression of recombinant antigen in *Escherichia coli* by sodium dodecyl sulfate extraction. Our study details a novel in silico approach for the development of flavivirus diagnostics, including a simple way to screen the important peptide regions.

## 1. Introduction

A majority of the viruses belonging to genus *Flavivirus* in family *Flaviviridae* are primarily spread through arthropod vectors (Arboviruses; arthropod-borne viruses). The Dengue virus (DENV), Japanese Encephalitis virus (JEV), Tick-borne Encephalitis virus (TBEV), West Nile virus (WNV), Yellow fever virus (YFV), and Zika virus (ZIKV) are all known to be prominent endemic human-pathogenic flaviviruses [1–3]. They cause illnesses ranging from mild symptoms to severe hemorrhagic shock syndrome, neurologic symptoms, and encephalitis [4]. The distribution of flaviviruses depends on the geographic location of their vectors and reservoirs, but their outbreaks are flaviviruses increasing worldwide, owing to climate change and subsequent habitat changes, as well as increased international travel [2, 5–7]. Flaviviruses are enveloped, positive-sense, single-stranded RNA viruses with an RNA genome that is organized to code three structural proteins and seven nonstructural (NS) proteins [8]. Among these, NS1, which provides an alternative approach for the differentiation of infectious flaviviruses, is a conserved NS protein across flaviviruses [9] and plays an important role in viral replication and eliciting host immune responses [10–14]. As secreted NS1 (sNS1) elicits an immune response, it can be used as a potential diagnostic marker for infections caused by flaviviruses. In addition, flavivirus NS1 has garnered much attention in the context of the development of subunit vaccines and therapeutics, owing to its importance in viral pathogenesis.

A large amount of genetic variation has been reported in flavivirus. A major problem in developing diagnostic biomarkers and vaccine candidates could be the high level of genetic and antigenic diversity in these viruses. A method for easy screening of viral antigenic diversity is required to address this problem. Even in the countries that currently do not face a flavivirus epidemic, the spread of mosquito-borne flaviviruses presents an increasing public threat, owing to climate change, international travel, and other factors. In Korea, there have been no reports of indigenous viral diseases, with the exception of JEV. However, the emergence of other medically important flaviviruses (such as DENV, WNV, YFV, and ZIKV) could present a significant threat in the future. However, in such countries, it is difficult to obtain resources such as various virus strains, in order to extract viral antigens, and there are major difficulties in controlling the spread of viruses, particularly those associated with imports. In this study, we propose that an *in silico* approach, using global databases and bioinformatics tools, can provide one of the alternative solutions.

Here, we attempted to process and analyze a large quantum of genetic variation (6,291) and antigen information from the global databases, GenBank and the Immune Epitope Database (IEDB). Using an in silico approach (phylogenetic analysis, frequency analysis, and the prediction of immunoepitopes) with easy-to-use shared and personal programs, we sought to propose a way for researchers to easily formulate a selection and design method. We also present a screening method using the *Escherichia coli* expression system to produce NS1 recombinant proteins from DENV, JEV, WNV, YFV, and ZIKV, based on selected candidates.

## 2. Materials and Methods

### 2.1. Retrieval of NS1 Protein Sequences from a Global Database, In Silico Design, and Gene Synthesis

**Figure 1:**
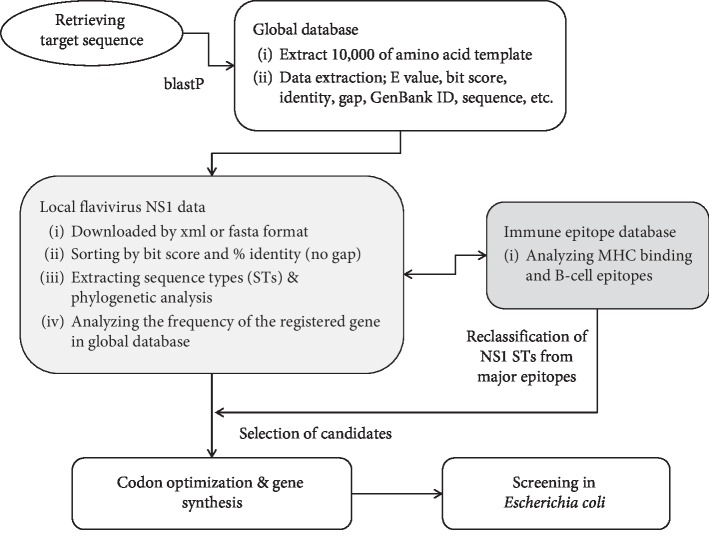
Overview of the in silico approach and the screening using the *Escherichia coli* expression system in this study.

NS1 amino acid sequences (352 amino acids) from the four serotypes of DENV (DENV1-4) and from JEV, WNV, YFV, and ZIKV were extracted from the NCBI GenBank by blastp using the position-specific iterated blast algorithm (https://blast.ncbi.nlm.nih.gov). These results (xml and fasta format files) were downloaded, converted into excel and fasta format files, and then filtered into a database. The sequence types were extracted according to their homology (considering the combination of identity and bit score in the result of blastp), followed by an analysis of their frequency, as registered on GenBank. These STs were analyzed using MegAlign Pro software (Dnastar Inc. Madison, WI, US) or the shared MEGA7 software, using Clustal and the Tamura-Nei model [15]. After alignment, the STs containing unspecified amino acid sequences (such as X) were removed. Phylogenetic analysis was then performed in MEGA7 software using Maximum Likelihood [16, 17]. In addition, antigen epitopes were investigated at the Immune Epitope Database and analysis resource (IEDB, http://www.iedb.org), and MHC binding predictions were made using the Consensus tool on April 1, 2019, based on a reference panel of 27 alleles [18–20]. The predicted epitopes were filtered according to the percentile rank for MHC class I binding or MHC class II binding by the consensus approach. B cell epitope prediction was performed using Bepipred Linear Epitope Prediction 2.0 [21]. We reclassified each STs by the predicted major epitopes and then selected the major STs of each flavivirus. All the variations of epitopes were assigned allele numbers and combined into an allelic profile and assigned an epitope type (EpT). Selected sequences among the STs were optimized for *E. coli* expression using the Codon Optimization On-Line tool (COOL, http://cool.syncti.org/) [22], which was followed by gene synthesis (Bioneer Corp., Daejeon, Korea). An overview of the entire process is presented in Figure 1.

### 2.2. Cloning and Expression of Recombinant NS1 Genes

*E. coli* strain Lemo21 (DE3) (New England BioLab Inc., Ipswich, MA, US) was used as the host for recombinant NS1 protein expression from DENV, JEV, WNV, YFV, and ZIKV. This NS1 gene was cloned downstream of a T7 promoter of the *E. coli* expression vector pBT7-N-His, to yield the plasmid pBT7-NS1. The resultant transformants were selected on Luria Bertani (LB) agar (Thermo Fisher Scientific, Waltham, MA, US) plates containing ampicillin (50 *μ*g/mL) (Sigma-Aldrich Korean Inc. Seoul, Korea) and chloramphenicol (30 *μ*g/mL) (Sigma-Aldrich Korean Inc.). Stock cultures of the microorganisms were maintained in LB broth with 30% glycerol (v/v) at −80°C. Plasmids of the positive transformants were extracted, and successful insertion was confirmed by sequence analysis.

Transformants confirmed by sequence analysis were further grown in LB medium at 35°C until mid-log phase. Protein expression was then induced by various concentrations of isopropyl *β*-D-1-thiogalactopyranoside (IPTG) (Sigma-Aldrich Korean Inc.) at various temperatures. One milliliter of each medium was harvested every hour to determine the optimal duration for high-level protein expression. The solubility of the produced recombinant proteins was assessed in each lysis buffer. Briefly, the cells were harvested at 5,000 rpm for 10 min at 4°C. The pellets were dissolved in lysis buffer containing 8 mM Na_2_HPO_4_, 286 mM NaCl, 1.4 mM KH_2_PO_4_, 2.6 mM KCl, 1 mM DTT, and 1% SDS (w/v) at pH 7.4 with lysozyme (1 mg/mL) and benzonase (nuclease inhibitor) (25 U/*μ*L) or native lysis buffer (without SDS), sonicated, and then centrifuged at 10,000 ×g for 30 min at 4°C to remove the cell debris. The supernatants were then analyzed by SDS-PAGE for expression and solubility.

### 2.3. Purification and Immunoblotting

For SDS lysis, the purification of each NS1 recombinant protein was performed as previously described [23]. Briefly, the samples were sonicated twice for 2 min each, using a microtip in a Misonic 3000 sonicator (Cole-Parmer North America, Vernon Hills, IL, US), set to 80% cycle and 40% power, at room temperature. After the ultracentrifugation of lysates, the supernatants were stored at 4°C for 2 h to precipitate the excess SDS, which was then removed by centrifugation and filtration through a 0.45 *μ*m syringe filter. Subsequently, Ni-NTA affinity purification was performed by column chromatography using Ni-NTA agarose (Qiagen, Manheim, Germany). Using a different concentration of imidazole, we purified the recombinant protein with a solution containing mild detergent (8 mM Na_2_HPO_4_, 286 mM NaCl, 1.4 mM KH_2_PO_4_, 2.6 mM KCl, and 0.1% Sarkosyl (w/v) at pH 7.4). Samples taken at various time points were analyzed by SDS-PAGE (4%–12% gradient gel) according to the manufacturer's recommendations and stained by Coomassie blue. Protein concentrations were estimated using the Pierce^®^ microplate BCA protein assay kit (Thermo Fisher Scientific).

The immunological properties of purified recombinant NS1 antigen were tested using western blot. The purified NS1 antigen was tested by anti-DENV and anti-ZIKV NS1 specific antibodies (The Native Antigen Company Ltd., Oxford, UK), and their cross-reactivity was checked and compared with control NS1 proteins from JEV, Tick-borne encephalitis virus (TBEV), WNV, YFV, and ZIKV (The Native Antigen Company Ltd.).

## 3. Results

### 3.1. Phylogenetic Analysis and Frequency Analysis

**Figure 2:**
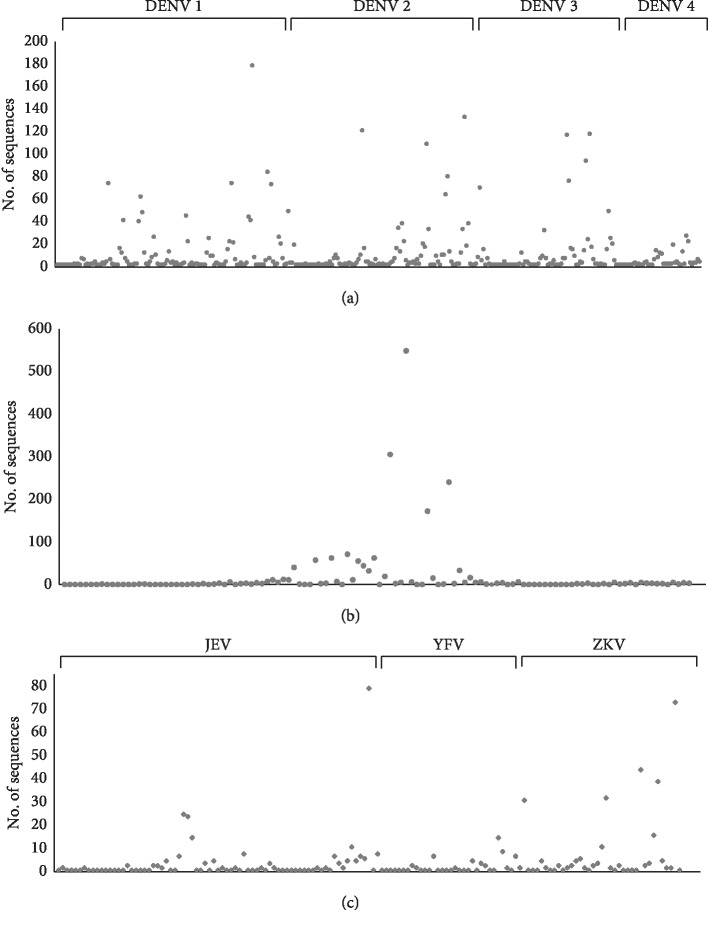
Frequency analysis of (a) DENV, (b) WNV (>200 of maximum frequency), and (c) JEV, YFV, and ZIKV (<200 of maximum frequency) NS1 genes. The *x*-axis indicates the sequence type (ST) of each flavivirus, while the *y*-axis represents the frequencies of each ST within the flavivirus NS1 genes registered in GenBank. These data were analyzed using blastp.

We analyzed several NS1 genes on GenBank, including 3,962 from DENV, 293 from JEV, 2,082 from WNV, 73 from YFV, and 413 from ZIKV. Among the GenBank registered genes, the frequencies of genes with 100% homology to each ST were analyzed by blastp (Figure 2). In Figure 2, each ST is arranged in such a manner that the genetic homology among STs is higher when the distance among STs is closer. For DENV-1 and DENV-2, the frequency of each ST was relatively broad compared with the other DENV types (Figure 2(a)). The frequency analysis of STs in WNV, YFV, and JEV revealed a comparatively clumped distribution around particular STs (Figures 2(b) and 2(c)).

**Figure 3:**
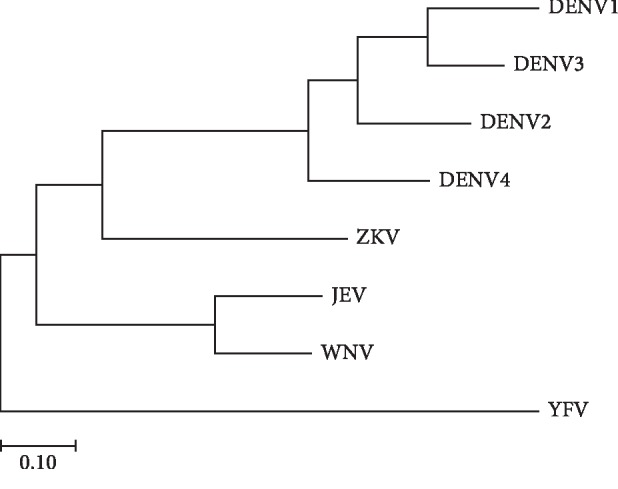
Phylogenetic analysis of the consensus sequence of each flavivirus NS1 using the Maximum Likelihood method, based on the JTT matrix-based model.

We performed the phylogenetic analysis of flaviviruses after frequency analysis. The genetic distance and phylogenetic analysis of each flavivirus NS1 consensus sequence revealed a molecular relationship and diversity in NS1 among flaviviruses (Figure 3). At this time, some sequences having an unspecified sequence (denoted by X) were removed, and we classified NS1 genes (amino acid sequence) having 100% homology as sequence type (ST).

Subsequently, 316 STs of DENV NS1 (homology 100%–67.0%), 59 STs of JEV (homology 100%–91.2%), 75 STs of WNV (homology 100%–86%), 43 STs of ZIKV (homology 100%–97%), and 30 STs of YFV (100%–92%) were extracted based on their homology. In addition, the interspecies phylogenetic analysis demonstrated that the genetic variants of DENV were clustered into six major groups (2 of type 1, 3 of type 2, 1 of type 3, and 1 of type 4), while JEV, WNV, YFV, and ZIKV were clustered into 2 groups each (see Figures [Supplementary-material supplementary-material-1]–[Supplementary-material supplementary-material-1] in the Supplementary Materials for comprehensive image analysis).

For the analysis of DENV, the unique ST (amino acid sequence) of DENV type 1 (DENV-1) was extracted from a total of 113 STs (the mean of genetic distance between STs = 0.0322). DENV-2 exhibited 94 STs (0.0494), DENV-3 showed 64 STs (0.0244), and DENV-4 had 45 STs (0.0527). In the case of JEV, the NS1 gene ST was primarily distributed in genotypes 1 and 3, with a lower distribution in genotypes 2, 4, and 5.

### 3.2. Prediction of MHC Binding Peptide in Flavivirus NS1 by an In Silico Approach

As the NS1 gene of flaviviruses was so genetically variable, it could be useful to reclassify them according to epitope types for the selection of recombinant protein candidates. Prior to reclassifying, we extracted the possible peptides corresponding to the T-cell response by the computational prediction analysis. For the prediction at IEDB, ∼18,000 peptides in MHC class I binding and ∼9,100 peptides in MHC class II binding were predicted from the consensus sequence of each flavivirus. The predicted peptides were then filtered at below 0.6 percentile rank (cut-off value, smaller values indicate better binding activity) for MHC class I binding or at below 1.0 percentile rank (cut-off value) for MHC class II binding, using the consensus approach. In addition, the core peptides were predicted by the combinatorial peptide library, SMM-align, and NN-align methods for the prediction of MHC class II binding peptide.

The prediction results for the MHC class II binding peptide in DENV NS1 revealed significant differences among serotypes, and even within each type, several variants were detected (Table 1). At below 1.0 percentile rank, there were four major regions in DENV-1 where the MHC class II binding core peptides were distributed. Seven such regions in DENV-2, four in DENV-3, and two in DENV-4 were predicted (Table 1). Also, eight MHC class I binding peptides in all serotypes were predicted as a common region (Table 2). Peptides at positions 28 and 48 in all serotypes exhibited a high affinity for MHC class II binding and lower variation than other regions of NS1, and in particular, common core peptides were detected between DENV-1 and DENV-3. Moreover, this region was predicted to exhibit similarity to that of the predicted MHC class I binding peptide (Table 2) and to B cell epitopes filtered at >0.4 percentile rank. As for the variation of core peptides within the same serotype, the peptides of DENV-2 appeared to be slightly more variable than those of other serotypes (Table 1). For the analysis of MHC class I binding, peptide ^26^HTWTEQYKF^34^ was conserved across all serotypes (Table 2). In addition, peptides ^20^FVTNEVHTW^28^, ^38^SPKRLSAAI^46^, and ^107^QPMEHKYSW^115^ were conserved between DENV-1 and DENV-3 (Table 2).

For the analysis of JEV genotypes, the most common genotypes were 1 and 3 in JEV infection, both of which exhibited nearly identical core peptide patterns for MHC II binding. However, these peptides did differ significantly from those of genotype 5 (Table 3). A similar pattern in the predicted core peptide at the N-terminus was observed among the genotypes, but there was a marked difference in those at the C-terminus (Table 3). Genotype 5 could function to predict the immune responses for other genotypes in JEV infection. A similar pattern in the predicted core peptide at the N-terminus of NS1 was also observed between JEV and WNV (Tables 3 and 4) as the analysis of WNV, YFV, and ZIKV indicated that there were almost no shared antigenic peptides for MHC class II binding (Table 4).

By the in silico searching, we investigated whether the predicted core peptides might be associated with any epitopes supported by some studies, including an *in vitro* T-cell response experiment. Except for a few, most core peptides in DENV, WNV, YFV, and ZIKV (Tables 1 and 4) were highly associated with the epitopes supported by the positive result of T-cell response (T-cell assay such as ELISPOT, ICS assay, and MHC ligand assay, etc.). These epitopes include all or part of the core peptides. There are their epitope IDs assigned at IEDB in Tables 1, 3, and 4. Only a predicted locus (amino acid pos. 192–211 in NS1) of JEV was associated with the reported MHC II epitopes, but others not (Table 3).

### 3.3. Reclassification of STs by the Major MHC II Binding Epitopes

STs were reclassified according to the variation of the major epitope to select candidates for the production of the recombinant NS1 protein from a large number of mutations. Among the entire NS1 amino acid sequences, we extracted the locus in which the core peptides (Tables 1–4) are distributed. All the variations of epitopes were assigned allele numbers and combined into an allelic profile and assigned an epitope type (EpT). 31 EpTs of DENV type 1 NS1 genetic variants, 33 EpTs of DENV type 2, 9 EpTs of DENV type 3, 5 EpTs of DENV type 5, 29 EpTs of JEV, 29 EpTs of WNV, 12 EpTs of YFV, and 5 EpTs of ZIKV were extracted according to their allelic profile (Table 5). There were dominant EpTs in all flaviviruses by the frequency analysis (Table 5). While there was one predominant EpT in most flaviviruses, two predominant EpTs were found in NS1 of DENV 2 and YFV. Based on the results of this study, we could select a major genotype for the production of the recombinant NS1 protein.

### 3.4. Screening of Flavivirus NS1 Recombinant Protein in the *E. coli* Expression System

Based on the genetic variation and frequency of each flavivirus NS1 ST, and the conserved epitopes and the discriminative epitopes among flaviviruses or serotypes, we were able to select candidates for the expression in the *E. coli* system. Eight candidate sequences were selected based on the frequency and the major STs in DENV NS1 (four of DENV-1, two of DENV-2, and one of DENV-3 and DENV-4 each). Four candidate sequences were selected based on the frequency and major genotypes in NS1 of JEV (two of genotype 2, and one of genotype 1 and genotype 3 each). In the case of WNV, YFV, and ZIKV, one candidate sequence each was selected, using the above methods.

A total of 15 NS1 genes (eight of DENV, four of JEV, and one of WNV, YFV, and ZIKV each) were optimized for *E. coli* cell expression and gene synthesis. The synthesized genes were cloned into a pBT7 expression vector. During screening for the expression and extraction of each NS1 recombinant protein, the solubility of all recombinant NS1 proteins in SDS was optimized for denaturing conditions during cell lysis. No optimization was carried out for native conditions (data not shown).

**Figure 4:**
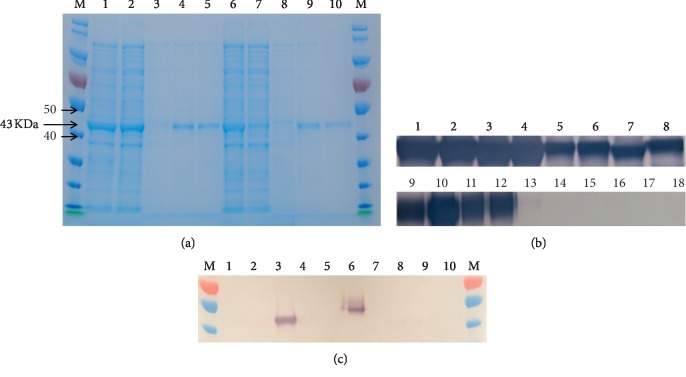
SDS polyacrylamide gel electrophoresis (PAGE) analysis showing the example of Ni-NTA purification of flavivirus NS1 recombinant protein by the *Escherichia coli* expression system (a), western blot analysis of DENV NS1 (b), and western blot analysis of ZIKV NS1 recombinant proteins (c). (a) Each fraction was loaded as follows: 1, *E. coli* lysate expressing DENV 1 NS1 recombinant protein after IPTG induction; 2, fraction (unbound) through-out in Ni-NTA column; 3, washing fraction at 50 mM imidazole; 4, elute 1 fraction at 150 mM imidazole; 5, elute 2 fraction at 150 mM imidazole; 6, *E. coli* lysate expressing DENV type 2 NS1 recombinant protein; 7, fraction (unbound) through-out in Ni-NTA column; 8, washing fraction; 9, elute 1 fraction; 10, elute 2 fraction, M: PageRuler protein ladder (Abcam plc., Cambridge, UK). (b) Anti-DENV NS1 primary antibody (titer 1 : 2,000) and anti-mouse IgG secondary antibody (1 : 20,000) were used. 1–4; DENV type 1 NS1 recombinant protein, 5-6; DENV type 2 NS1 recombinant protein, 7-8; DENV type 3 and 4 NS1 recombinant protein, 9–12; DENV type 1–4 NS1 recombinant protein (Native Antigen Company), 13; JEV-NS1 (Native Antigen Company), 14; TBEV NS1 (Native Antigen Company), 15; WNV-NS1 (Native Antigen Company), 16; YFV-NS1 (Native Antigen Company), 17; ZIKV Uganda strain NS1 (Native Antigen Company), 18; ZIKV Suriname strain NS1 (Native Antigen Company). (c) Anti-ZIKV NS1 primary antibody (titer 1 : 2,000) and anti-mouse IgG secondary antibody (1 : 20,000) were used. 1; WNV NS1 recombinant protein, 2; YFV NS1 recombinant protein, 3; ZIKV NS1 recombinant protein, 4; JEV NS1 recombinant protein, 5; DENV NS1 recombinant protein, 6; ZIKV Uganda strain NS1 (Native Antigen Company), 7; DENV type 1 NS1 (Native Antigen Company), 8; DENV type 2 (Native Antigen Company), 9; DENV type 3 (Native Antigen Company), 10; DENV type 4 (Native Antigen Company), M; PageRuler protein ladder (Abcam).

The NS1 diagnostic antigen (approximately 43 kDa) was found to be overexpressed at 3 h (T3) after IPTG induction, but the expression level began to decrease significantly after 4 h. Instead of urea, we used a lysis buffer containing high concentrations of SDS (34 mM, 1% w/v), which could dissolve aggregates of NS1 recombinant proteins upon sonication. The lysates were then cooled to precipitate the excess SDS. SDS was replaced by the mild detergent sarkosyl (0.1%, w/v) during Ni-NTA chromatography. All hexahistidine-tagged NS1 proteins were eluted using 150 mM imidazole (Figure 4(a)). We observed that the combined flow-through and wash fraction contained only small amounts of the unbound fusion protein.

A total of eight DENV NS1 recombinant antigens specifically reacted with the anti-DENV NS1 IgG monoclonal antibodies (the native antigen) compared with other flaviviruses (control antigen of JEV, TBEV, WNV, YFV, and ZIKV NS1 antigens) (Figure 4(b)). Moreover, the NS1 recombinant protein from ZIKV developed in this study revealed high immunoreactivity against the anti-ZIKV NS1 IgG monoclonal antibody during western blot analysis (Figure 4(c)).

## 4. Discussion

For serological diagnosis of flavivirus infection, measurement of antibodies specific to the envelope (E) proteins and premembrane (prM) is used as the front-line assay; however, cross-reactivity has been reported among flavivirus infections owing to similarity in antigenic epitopes [24–27], which complicates the interpretation of diagnosis results. Therefore, alternative approaches are required to address these cross-reactivity problems. NS1 from flaviviruses provides an important candidate biomarker for the development of diagnostics, therapeutics, and vaccines, as well as the assessment of the pathogenesis of these viruses. Measurement of specific antibody responses to NS1 using IgM and IgG antibody capture enzyme-linked immunosorbent assays (MAC- and GAC-ELISAs) could provide an alternative approach for the identification of infectious flaviviruses [28–30]. In addition, it has been reported that anti-JEV NS1 antibodies can function as serological markers of natural infection within the vaccinated population [31, 32], and anti-DENV NS1 IgG ELISA can detect previous dengue exposure prior to vaccination [33]. In particular, the detection of previous exposure and quantification of the serostatus is important prior to vaccination, as flavivirus cross-reactive antibodies may contribute to the severity of secondary flavivirus infection, owing to antibody-dependent enhancement (ADE) [34].

There are numerous existing genetic variants of flaviviruses, while new viral strains also keep emerging, owing to their adaptability to the environment and the increased vector distribution and transmission by climate change. Therefore, it is urgent that the medical community understands the magnitude of the genetic variation in flaviviruses, to cope with the emerging variants. In this context, we aimed to analyze the NS1 genetic and antigenic variants of endemic flaviviruses (DENV, JEV, WNV, YFV, and ZIKV) in a global database (GenBank and IEDB) and design genetic synthetic NS1 antigens. Initially, 316 NS1 STs from DENV, 59 from JEV, 75 from WNV, 43 from ZIKV, and 30 from YFV were selected from 6,823 NS1 sequences registered in GenBank and analyzed phylogenetically (see Figures [Supplementary-material supplementary-material-1]–[Supplementary-material supplementary-material-1] in the Supplementary Materials for comprehensive image analysis). For the frequency analysis of each ST, a comparatively clumped distribution was found around particular STs in WNV, YFV, and JEV, indicating that closely genetically related STs are the dominant distribution type (Figure 2). However, it is likely that genetically diverse strains could be distributed in DENV, as the frequency of DENV STs was relatively broad compared with that of other flaviviruses (Figure 2). These results detailing the phylogenetic relationship and the frequency of particular genetic variants could be used as the primary criterion for selecting a specific genotype from a large number of genetic variants, to design a diagnostic biomarker or vaccine candidate.

The filtered STs, however, were still too numerous, even after the analysis of the genetic variation and frequency, and the selection criteria were vague and complicated. Therefore, we investigated the antigenic variation and differences among endemic flaviviruses from the IEDB database and assessed the antigenic variation of STs (Tables 1–4). Upon retrieval of each NS1 consensus sequence for the human MHC class II binding site, we could analyze the characteristics of the predicted, highly antigenic core peptide in each flavivirus, as well as determining the differences and similarity among serotypes or species, along with the variation within the same serotypes or species. These data could be of substantial utility during designing of antigens for discrimination among flaviviruses, as they could predict the CD4^+^ or/and CD8^+^ T-cell response. For example, peptide ^27^TWTEQYKF^34^ in all DENV types was predicted to show MHC class I and II binding (Tables 1 and 2). JEV and WNV, members of the same serocomplex, shared certain core peptides such as RCGSGVF and HNDVEAWM at the N-terminus (Tables 3 and 4). However, prediction analysis possibly picks up peptides that are not actually bound to the MHC molecule (false positive). Also, the proven epitopes for T-cell response could be ruled out according to the setting of the cut-off value or other algorithms (false negative). The prediction analysis by the computational method should be supported or compared by actual experimental results. In our study, the predicted peptides ^38^SPKRLSAAI^36^ (IEDB epitope ID no. 60104), ^26^HTWTEQYKF^34^ (no. 183619), and ^31^QYKFQADSPKRLSAA^45^ in DENV (Tables 1 and 2) were studied for their immune reactivity, with positive results obtained from T-cell assays and MHC ligand assays.

But it was discussed that the CD8^+^ T-cell response by the NS1 peptides is very low [35]. Otherwise, Rivino et al. [36] showed the CD4^+^ T-cell response by the epitopes of NS1 by ELISPOT and ICS assay. CD4^+^ T cells could elicit protective antibody responses and generate both B-cell and CD8^+^ T-cell memory responses during the viral infection. Also, they showed that the dengue virus-specific CD4^+^ T cell has the phenotype of circulating follicular helper T cells which could interact with B cells [36]. As NS1 has CD4^+^ epitopes and is also targeted by B lymphocytes, we presumed that it might be effective for reclassifying NS1 types according to CD4^+^ epitopes. When retrieved on the IEDB, most core peptides in DENV, WNV, YFV, and ZIKV (Tables 1 and 4) were highly associated with the epitopes supported by the positive result of T-cell response (T-cell assay such as ELISPOT, ICS assay, and MHC ligand assay). Taken together, we reclassified STs according to the variation of the major epitope by MHC II binding. 78 DENV epitope type (EpT), 29 JEV EpTs, 29 WNV EpTs, 12 YFV EpTs, and 5 ZIKV EpTs were extracted (Table 5). The reclassification of many STs by the locus of major epitopes and frequency analysis showed that there could be predominant epitope types as well as many EpTs with the diverse epitope in the NS1 gene of each flavivirus (Table 5). These results could provide a simple way for the selection of candidates for the production of the recombinant protein.

When the genetic variants and antigenic epitopes are known, NS1 in each flavivirus could be classified to select candidates for recombinant protein production. During the screening for recombinant protein expression in *E. coli*, 15 different NS1 recombinant proteins were successfully expressed under mild denaturing conditions (SDS). Among these, the NS1 recombinant proteins from DENV and ZIKV were determined to be immunoreactive, when compared with human expressed recombinant proteins, which were used as controls (Figure 4). This immunoreactivity, however, should be further examined using clinical specimens. In this study, each NS1 recombinant protein was screened using *E. coli* expression, considering the convenience and cost factors. However, there could be some limitations in the recombinant proteins produced by the prokaryotic expression, such as differences in biological activity, posttranslational processing, or glycosylation. The immunoreactivity should be further studied and compared with that of the recombinant NS1 protein produced by other eukaryotic expression systems. Nevertheless, the NS1 data established in this study can also be applied as a template for eukaryotic expression, to address the limitations of prokaryotic expression of recombinant proteins. Finally, this approach can also be applied to genetic design, allowing the avoidance of conserved epitopes or the investigation of other pathogens.

## 5. Conclusions

This study reported a simple in silico approach, easily accessible to individual researchers (using the shared and personal programs), for the large-scale examination of conserved and variable sequences in endemic flaviviruses from a global database. Our results demonstrated the possibility of in silico designing of viral recombinant antigens for the identification of serotypes or species, thereby avoiding the use of conserved epitopes.

## Figures and Tables

**Table 1:** Prediction of MHC class II binding peptide in DENV NS1 using the T-cell epitope prediction tool at IEDB.

	Amino acid position^a^	Human allele	Mean percentile rank^b^	Mean IC_50_^c^	Core peptide^d^	Homology (%)^e^	Epitope ID^f^
DENV-1	28–48	HLA-DRB	0.207	49	**WTEQYKFQA**	99.56	869135 132583
					**TEQYKFQAD**	99.56	
					**QYKFQADSP**	99.56	
					**FQADSPKRL**	99.44	
					SPKRLSAAI	98.89	
	95–113	HLA-DRB	0.228	60.6	ILAQGKKMI	96.89	738503
					GKKMIRPQP	98.11	
					KKMIRPQPM	98.38	
	157–175	HLA-DPA/DPB	0.574	79.22	GFGIFTTNI	98.33	869721 866693
					IFTTNIWLK	98.22	
					**FTTNIWLKL**	99.44	
	204–224	HLA-DRB	0.217	45.64	SEKNETWKL	99.0	195579 195784
					NETWKLARA	98.67	
					ETWKLARAS	98.78	
					TWKLARASF	99.22	
					WKLARASFI	99.44	
					KLARASFIE	99.44	

DENV-2	14–33	HLA-DRB	0.4	64.5	KCGSGIFIT	96.67	
					IFITDNVHT	96.33	
					FITDNVHTW	96.22	
	28–43	HLA-DRB	0.65	146	TEQYKFQPE	98.56	539828
					YKFQPESPS	98.33	
					FQPESPSKL	98.67	
	157–176	HLA-DPA/DPB	0.614	118.25	GVFTTNIWL	98.11	866460
					VFTTNIWLK	96.11	869722
					**FTTNIWLKL**	97.33	866831 866699
	192–211	HLA-DQA/DQB	0.215	456.83	DMGYWIESA	98.44	867040
					MGYWIESAL	98.11	
	204–223	HLA-DRB	0.062	595.67	SALNDTWKM	94.33	539079 539708
					TWKMEKASF	96.0	
					WKMEKASFI	96.11	
	229–244	HLA-DQA/DQB	0.88	542.5	WSNGVLESE	99.22	
					GVLESEMII	98.11	
	317–335	HLA-DRB	0.46	415.8	TLPPLRYRG	99.11	180396
					YRGEDGCWY	99.22	
					RGEDGCWYG	99.5	
					GEDGCWYGM	99.67	
					EDGCWYGME	99.78	
					DGCWYGMEI	100	

DENV-3	28–48	HLA-DRB	0.435	48.15	**WTEQYKFQA**	99.56	190996 539825 196235 190810
					**TEQYKFQAD**	99.56	
					**QYKFQADSP**	99.56	
					**FQADSPKRL**	99.56	
					SPKRLATAI	99.78	
	157–176	HLA-DPA/DPB	0.703	128.15	GVFTTNIWL	99.44	866460 869722 866831 866700
					VFTTNIWLK	99.11	
					**FTTNIWLKL**	99.11	
	192–210	HLA-DQA/DQB	0.702	819.6	DMGYWIESQ	99.78	190715 867042
	206–223	HLA-DRB	0.806	78.11	SWKLEKASL	97.56	539717 195419
					WKLEKASLI	97.56	

DENV-4	14–34	HLA-DRB	0.193	52.86	KCGSGIFVV	95.11	46388 183304 183696
					IFVVDNVHT	95.11	
					FVVDNVHTW	95.33	
					HTWTEQYKF	99.78	
					**TEQYKFQPE**	99.78	
	28–46	HLA-DRB	0.282	95.6	YKFQPESPA	99.56	539829
					FQPESPARL	99.56	
					QPESPARLA	99.56	

These data were extracted by filtering at below 1.0 percentile rank. Bold letters indicate the same core peptide among DENV serotypes. ^a^NS1 amino acid sequence number. ^b^Mean of percentile ranks of the predicted peptides in this region. ^c^Mean of IC_50_ by SMM-align method for the predicted peptides in this region. ^d^Data predicted by the combinatorial peptide library, SMM-align, and NN-align methods. ^e^Values calculated as mean of the homology for each amino acid sequence of each ST. ^f^These represent epitopes associated with the positive result of T-cell response (T-cell assay such as ELISPOT, ICS assay, and MHC ligand assay), previously reported at some reference. Epitope ID is assigned at IEDB. These epitopes include all or part of the core peptides in the table.

**Table 2:** MHC class I binding peptide common in DENV types predicted by the T-cell epitope prediction tool at IEDB.

Start no. of amino acid position	Major allele	Peptide of DENV-1	Peptide of DENV-2	Peptide of DENV-3	Peptide of DENV-4
20	HLA-B*∗*53:01	**FVTNEVHTW**	FITDNVHTW	**FVTNEVHTW**	FVVDNVHTW
26	HLA-A*∗*32:01	H**TWTEQYKF**	H**TWTEQYKF**	H**TWTEQYKF**	H**TWTEQYKF**
38	HLA-B*∗*07:02	**SPKRLSAAI**	SPSKLASAI	**SPKRLATAI**	SPARLASAI
107	HLA-B*∗*53:01	**QPMEHKYSW**	QPTELKYSW	**QPMELKYSW**	PASDLKYSW
160	HLA-B*∗*58:01	FGIFTTNIW	**FGVFTTNIW**	**FGVFTTNIW**	FGMFTTNIW
243	HLA-B*∗*07:02	IPKIYGGPI	IPKNFAGPV	IPKSLAGPI	IPKSYAGPF
252	HLA-B*∗*15:01	SQHNYRPGY	SQHNYRPGY	SQHNHRPGY	SQHNYRQGY
303	HLA-B*∗*58:01	VTGKIIHEW	ASGKLITEW	VSGKLIHEW	ASGKLVTQW

These data were extracted by filtering at below 0.6 percentile rank. Bold letters indicate that the peptide is shared with the MHC class II binding core peptide.

**Table 3:** Prediction of MHC class II binding peptides in JEV NS1 by the T-cell epitope prediction tool at IEDB.

Genotype	Amino acid position^a^ (epitope ID)^d^	Human allele	Mean percentile rank^b^	Core peptide^c^
Genotype 1 & 3	14–32	HLA-DRB	0.568	**RCGSGIFVH** **FVHNDVEAW** **VHNDVEAWV**
	91–109	HLA-DRB	0.474	RYRSAPKRL YRSAPKRLS
	143–159	HLA-DQA/DQB	0.687	CPDEHRAWN EHRAWNSMQ **AWNSMQIED** **WNSMQIEDF** NSMQIEDFG
	148–168	HLA-DRB	0.317	**WNSMQIEDF** **QIEDFGFGI** **FGITSTRVW**
	192–211 (539105, 540128)	HLA-DQA/DQB	0.502	DLSYWIESR LSYWIESRY
	267–287	HLA-DRB, HLA-DQA/DQB	0.116	WDENGIVLD ENGIVLDFD IVLDFDYCP
	317–335	HLA-DRB	0.698	SLPPLRFRT RFRTENGCW FRTENGCWY
	332–350	HLA-DRB	0.316	GMEIRPVRH VRHDETTLV

Genotype 5	14–32	HLA-DRB	0.568	**RCGSGIFVH** **FVHNDVEAW** **VHNDVEAWV**
	144–158	HLA-DQA/DQB	0.95	**AWNSMQIED** **WNSMQIEDF**
	148–168	HLA-DRB	0.284	**WNSMQIEDF** **QIEDFGFGI** **FGITSTRVW**
	192–211 (539105, 540128)	HLA-DQA/DQB	0.195	DLSYWIESH LSYWIESHL
	267–287	HLA-DRB	0.171	DENEITLDF ITLDFDYCP FDYCPGTTV
	332–349	HLA-DRB	0.458	GMEIRPMKH IRPMKHDES MKHDESTLV

These data were extracted by filtering at below 1.0 percentile rank. Bold letters indicate the same core peptide among JEV genotypes. ^a^NS1 amino acid sequence number. ^b^Mean of percentile ranks of the predicted peptides in this region. ^c^Values predicted by the combinatorial peptide library, SMM-align, and NN-align methods. ^d^These represent epitopes associated with the positive result of T-cell response (T-cell assay such as ELISPOT, ICS assay, and MHC ligand assay), previously reported at some reference. Epitope ID is assigned at IEDB. These epitopes include all or part of the core peptides in the table.

**Table 4:** Prediction of MHC class II binding peptide in NS1 from WNV, YFV, and ZIKV by the T-cell epitope prediction tool at IEDB.

Amino acid position^a^ (epitope ID)^d^	Human allele	Mean^b^	Core peptide^c^
*WNV*			
14–32	HLA-DRB	0.642	**RCGSGVF**I**H** **F**I**HNDVEAW** I**HNDVEAWM**
91–109	HLA-DRB	0.102	MYK**SAPKRL** YK**SAPKRL**T
144–158	HLA-DQA/DQB	0.96	QNRAWNSLE AWN**S**LEVED
154–174 (21175)	HLA-DRB, HLA-DPA/DPB	0.611	EVEDFGFGL FGLTSTRMF GLTSTRMFL LTSTRMFLK TSTRMFLKV
188–211 (159026)	HLA-DRB, HLA-DQA/DQB	0.244	AIHSDLSYW IHSDLSYWI **DLSYWIESR** **LSYWIESR**L
317–336 (57994)	HLA-DRB	0.293	YQTDSGCWY TLPPLRYQT

*YFV*			
15–31 (10088)	HLA-DQA/DQB	0.91	IFIFRDSDD IFRDSDDWL
64–78 (113828)	HLA-DRB, HLA-DQA/DQB	0.162	EHEMWRSRA SRADEINAI ADEINAIFE DEINAIFEE
71–95 (113319, 137424)	HLA-DQA/DQB	0.633	DEINAIFEE NAIFEENEV AIFEENEVD FEENEVDIS EENEVDISV EVDISVVVQ VDISVVVQD DISVVVQDP
144–158 (181115]	HLA-DPA/DPB, HLA-DQA/DQB	0.804	NRVWNSFQI RVWNSFQIE SFQIEEFGT FQIEEFGTG RVYMDAVFE VYMDAVFEY
169–183 (137404, 232424]	HLA-DRB	0.01	MDAVFEYTI YTIDCDGSI
207–221	HLA-DQA/DQB	0.7	WMIHTLEAL
222–237 (231830152485)	HLA-DRB	0.675	KECEWPLTH LTHTIGTSV

*ZIKV*			
14–31 (569047, 863166, 871319)	HLA-DRB	0.89	RCGTGVFVY FVYNDVEAW YNDVEAWRD
28–46 (862875, 862438)	HLA-DRB	0.69	WRDRYKYHP RDRYKYHPD YHPDSPRRL
67–86 (862872, 862852)	HLA-DQA/DQB	0.57	SVEGELNAI VEGELNAIL EGELNAILE GELNAILEE ELNAILEEN NAILEENGV
142–160 (862457, 862672, 863124]	HLA-DPA/DPB	0.321	HRAWNSFLV RAWNSFLVE
157–160 (862889, 862596]	HLA-DPA/DPB	0.819	GVFHTSVWL VFHTSVWLK FHTSVWLKV HTSVWLKVR
193–201 (862673]	HLA-DQA/DQB	0.668	DLGYWIESE

These data were extracted by filtering at below 1.0 percentile rank. Bold letters indicate the same core peptide as that of JEV. ^a^NS1 amino acid sequence number. ^b^Mean of percentile ranks of the predicted peptides in this region. ^c^Values predicted by the combinatorial peptide library, SMM-align, and NN-align methods. ^d^These represent epitopes associated with the positive result of T-cell response (T-cell assay such as ELISPOT, ICS assay, and MHC ligand assay), previously reported at some reference. Epitope ID is assigned at IEDB. These epitopes include all or part of the core peptides in the table.

**Table 5:** The reclassification of each STs by the locus of major epitopes.

	DENV1	DENV2	DENV3	DENV4	JEV	WNV	YFV	ZIKV
Major epitope locus^a^	E1 (28–46)	E1 (14–43)	E1 (28–46)	E1 (14–43)	E1 (14–29)	E1 (14–29)	E1 (19–29)	E1 (14–42)
	E2 (96–109)	E2 (161–171)	E2 (161–171)		E2 (96–105)	E2 (96–105)	E2 (64–93)	E2 (70–84)
	E3 (159–171)	E3 (197–218)	E3 (197–205)		E3 (143–168)	E3 (146–171)	E3 (147–183)	E3 (147–172)
	E4 (204–219)	E4 (232–243)	E4 (209–218)		E4 (197–206)	E4 (193–205)	E4 (211–236)	E4 (197–205)
		E5 (317–335)			E5 (268–281)	E5 (317–331)		
					E6 (317–346)			

ST^b^	113	94	64	45	59	75	30	43

EpT^c^	31	33	9	5	29	29	12	5

Frequency of major EpT (allelic profile) (%)^d^	EpT 1 (1-1-1-1) (73.9%)	EpT 1 (1-1-1-1-1) (31.5%)	EpT 1 (1-1-1-1) (84.3%)	EpT 1(1) (67.7%)	EpT 1 (1-1-1-1-1-1) (37.9%)	EpT 1 (1-1-1-1-1) (78.6%)	EpT 1 (1-1-1-1) (23.3%)	EpT 1 (1-1-1-1) (88.4%)
	EpT 2 (1-6-5-1) (10.1%)	EpT 2 (1-1-3-1-1) (38.2%)	EpT 2 (1-1-1-1) (13.3%)	EpT 1(2) (24.4%)	EpT 1 (1-1-2-3-1-1) (30.7%)		EpT 2 (1-4-1-1) (20.6%)	
	EpT 3 (1-1-1-2) (5.9%)							

^a^Major epitope locus is the region in which the core peptides (Tables 1–4) are distributed. The number in parentheses is the amino acid sequence number. ^b^Sequence type (ST) was classified by the phylogenetic analysis. The numbers in this row represent the total sum of each ST. ^c^All the variations of epitopes were assigned allele numbers and combined into an allelic profile and assigned an epitope type (EpT). The numbers in this row represent the total sum of each EpT. ^d^The frequency registered on GenBank.

## Data Availability

The data used to support the findings of this study are available from the corresponding author upon request.

## Supplementary Materials

Supplementary Materials Figure S1: phylogenetic analysis of DENV type 1 NS1 STs by Maximum Likelihood method based on the JTT matrix-based model. Figure S2: phylogenetic analysis of DENV type 2 NS1 STs by Maximum Likelihood method based on the JTT matrix-based model. Figure S3: phylogenetic analysis of DENV type 3 NS1 STs by Maximum Likelihood method based on the JTT matrix-based model. Figure S4: phylogenetic analysis of DENV type 4 NS1 STs by Maximum Likelihood method based on the JTT matrix-based model. Figure S5: phylogenetic analysis of JEV NS1 STs by Maximum Likelihood method based on the JTT matrix-based model. Figure S6: phylogenetic analysis of WNV NS1 STs by Maximum Likelihood method based on the JTT matrix-based model. Figure S7: phylogenetic analysis of YFV NS1 STs by Maximum Likelihood method based on the JTT matrix-based model. Figure S8: phylogenetic analysis of ZIKV NS1 STs by Maximum Likelihood method based on the JTT matrix-based model.
