# Supplementary material for: A Simple Method for the Design and Development of Flavivirus NS1 Recombinant Proteins Using an In Silico Approach
Source: Biomed Res Int. 2020 Feb 13;2020:3865707. doi: 10.1155/2020/3865707 (PMC7040382; doi:10.1155/2020/3865707)
Supplement: Supplementary Materials — Figure S1: phylogenetic analysis of DENV type 1 NS1 STs by Maximum Likelihood method based on the JTT matrix-based model. Figure S2: phylogenetic analysis of DENV type 2 NS1 STs by Maximum Likelihood method based on the JTT matrix-based model. Figure S3: phylogenetic analysis of DENV type 3 NS1 STs by Maximum Likelihood method based on the JTT matrix-based model. Figure S4: phylogenetic analysis of DENV type 4 NS1 STs by Maximum Likelihood method based on the JTT matrix-based model. Figure S5: phylogenetic analysis of JEV NS1 STs by Maximum Likelihood method based on the JTT matrix-based model. Figure S6: phylogenetic analysis of WNV NS1 STs by Maximum Likelihood method based on the JTT matrix-based model. Figure S7: phylogenetic analysis of YFV NS1 STs by Maximum Likelihood method based on the JTT matrix-based model. Figure S8: phylogenetic analysis of ZIKV NS1 STs by Maximum Likelihood method based on the JTT matrix-based model. [file 3865707.f1.pptx]

## Slide 1
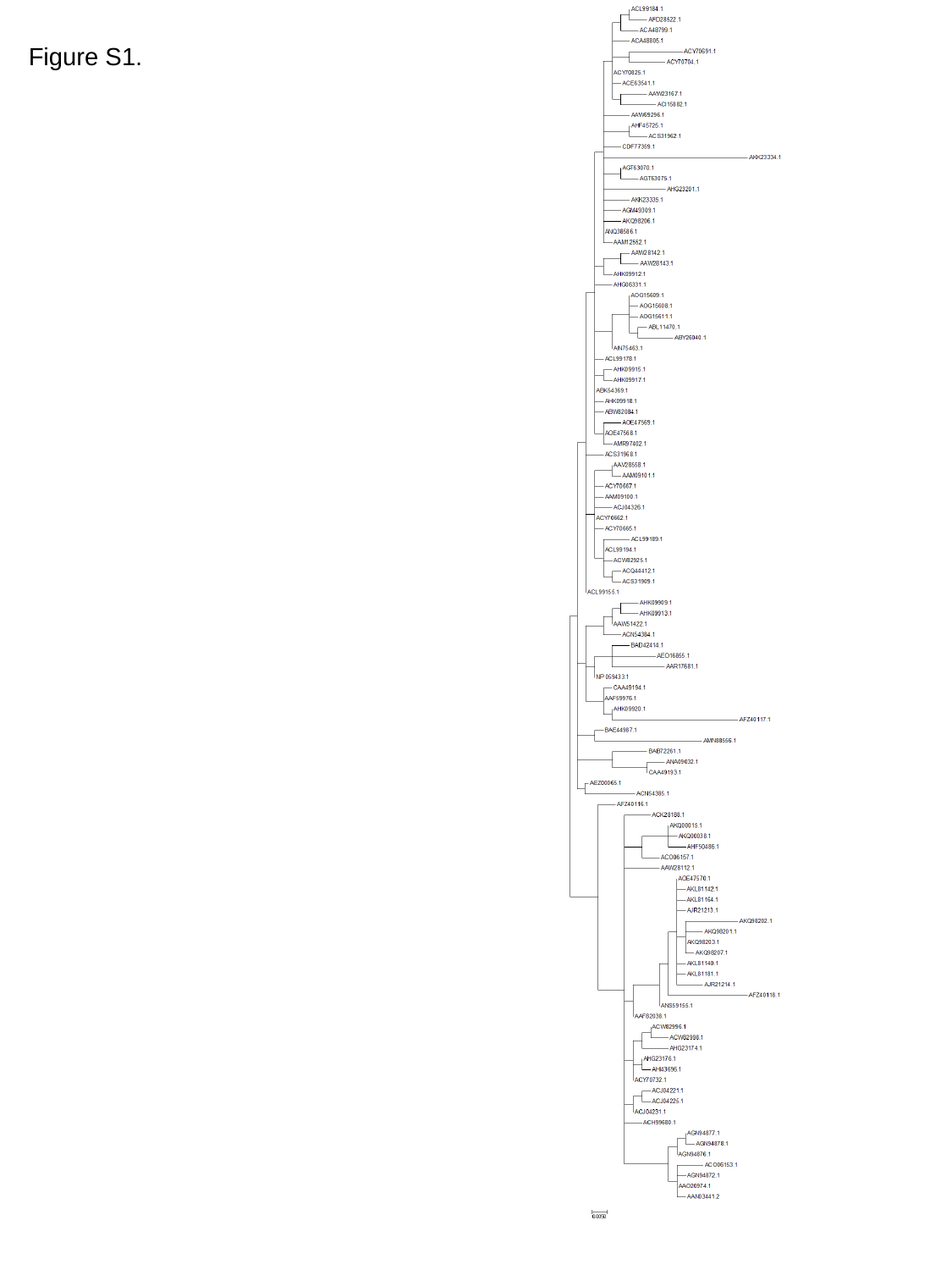

Figure S1.

## Slide 2
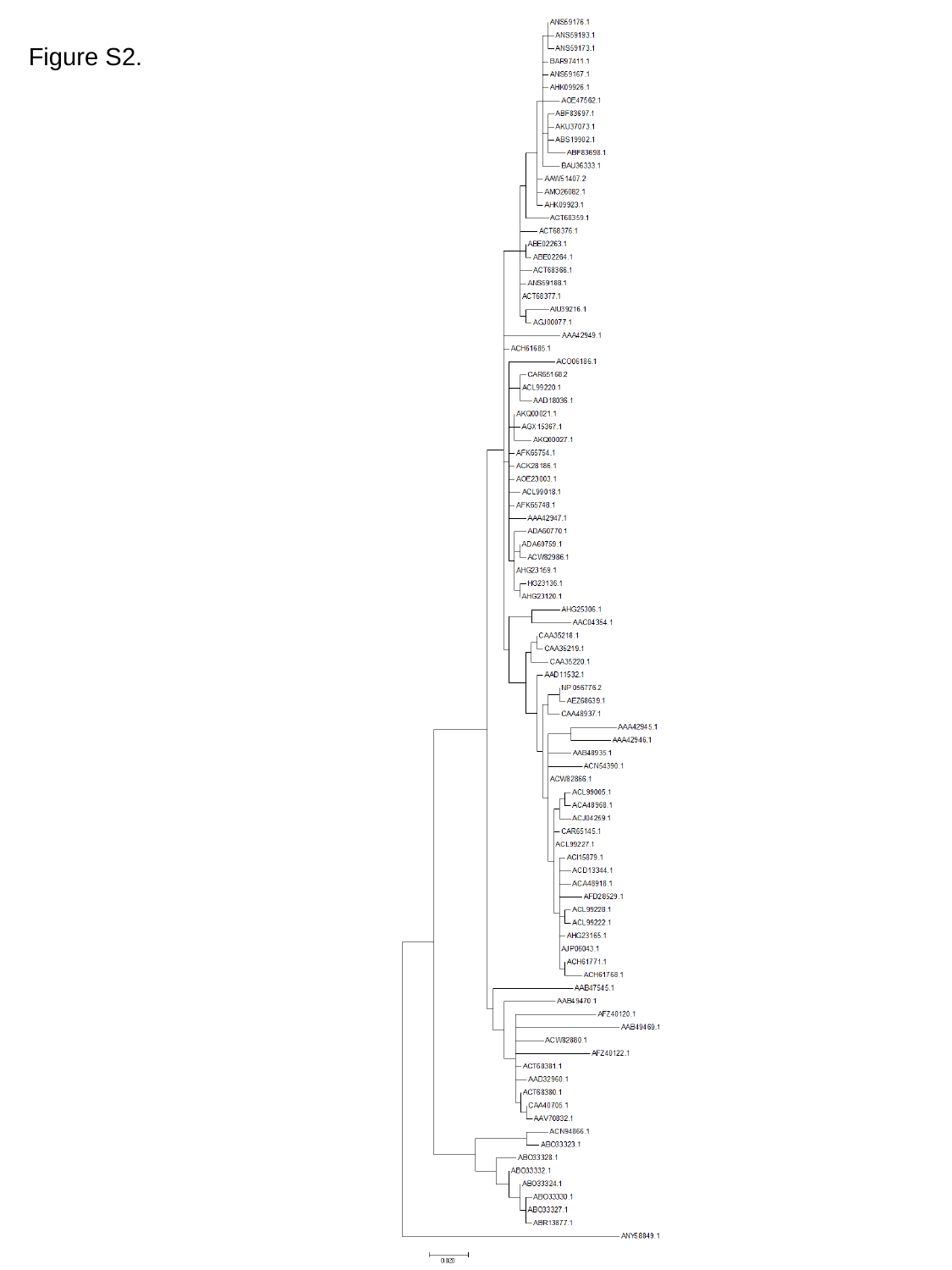

Figure S2.

## Slide 3
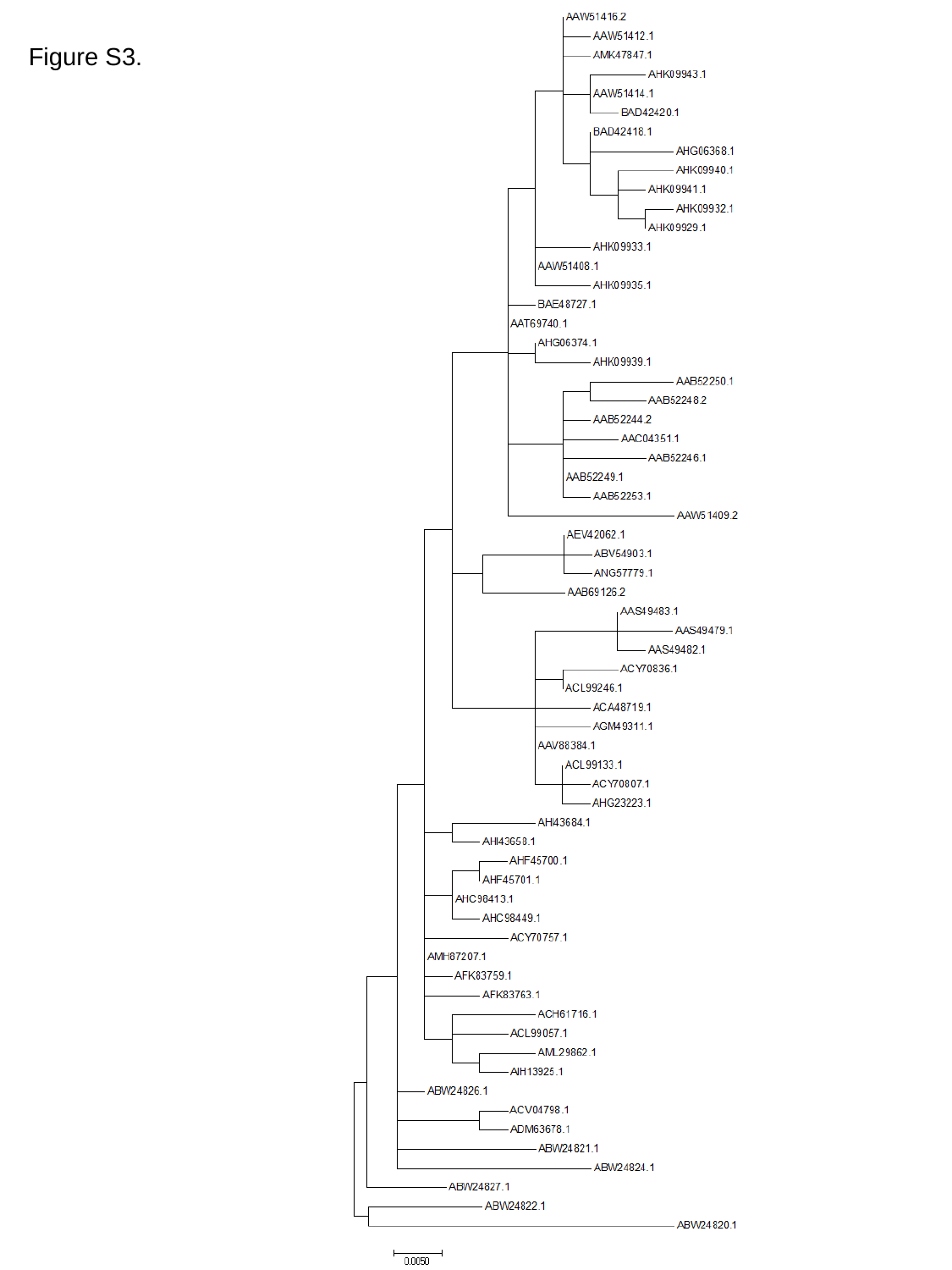

Figure S3.

## Slide 4
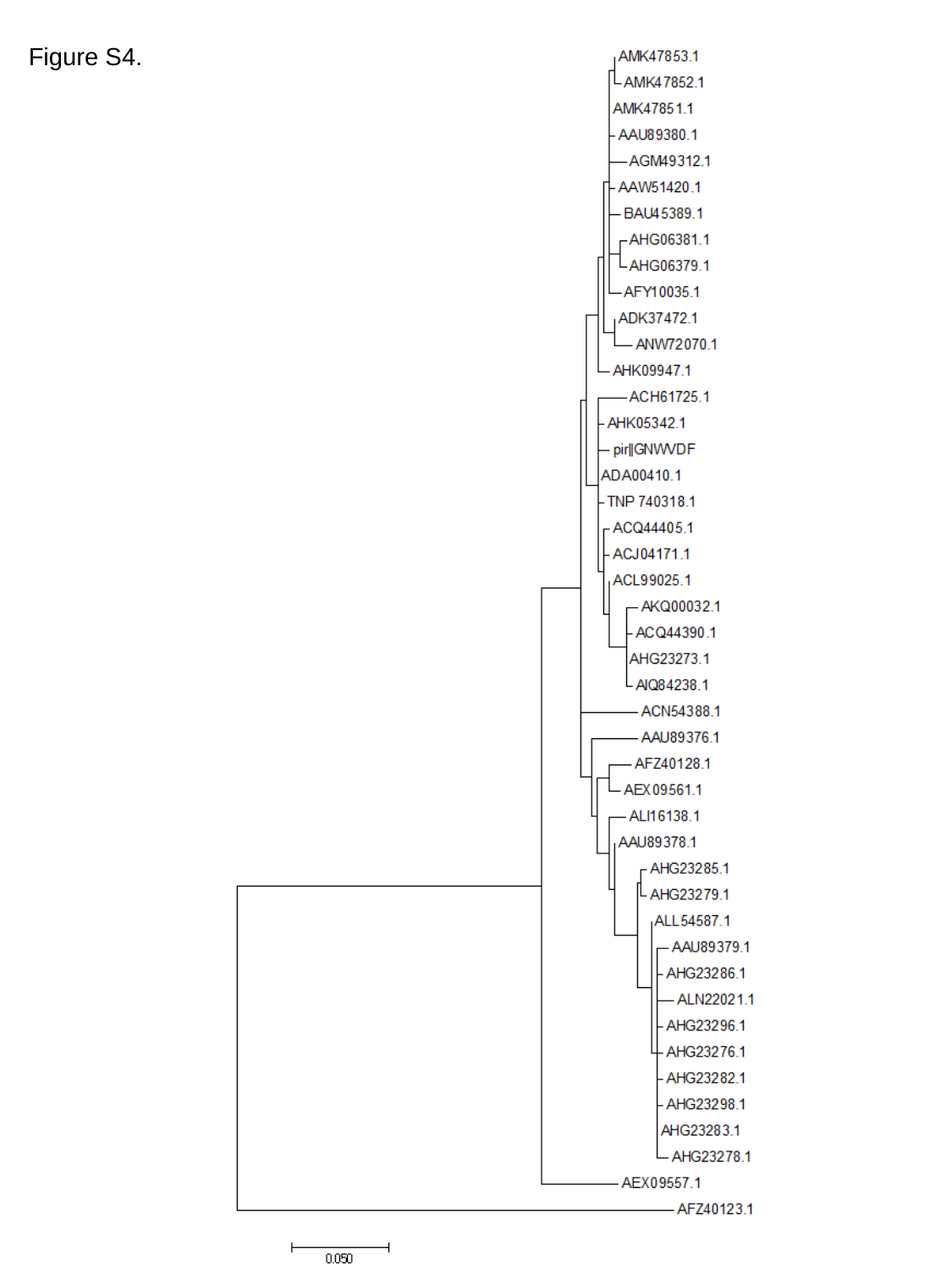

Figure S4.

## Slide 5
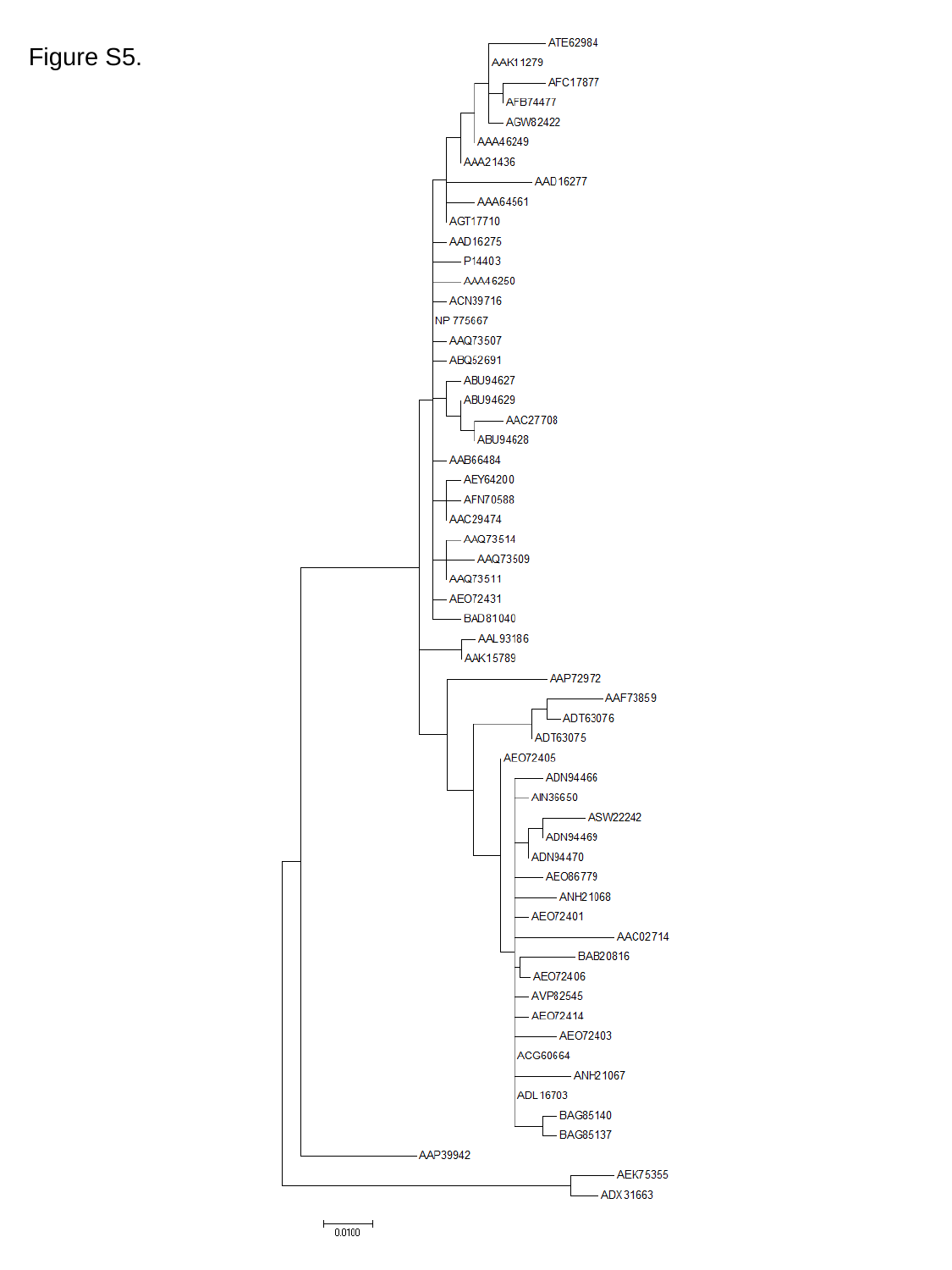

Figure S5.

## Slide 6
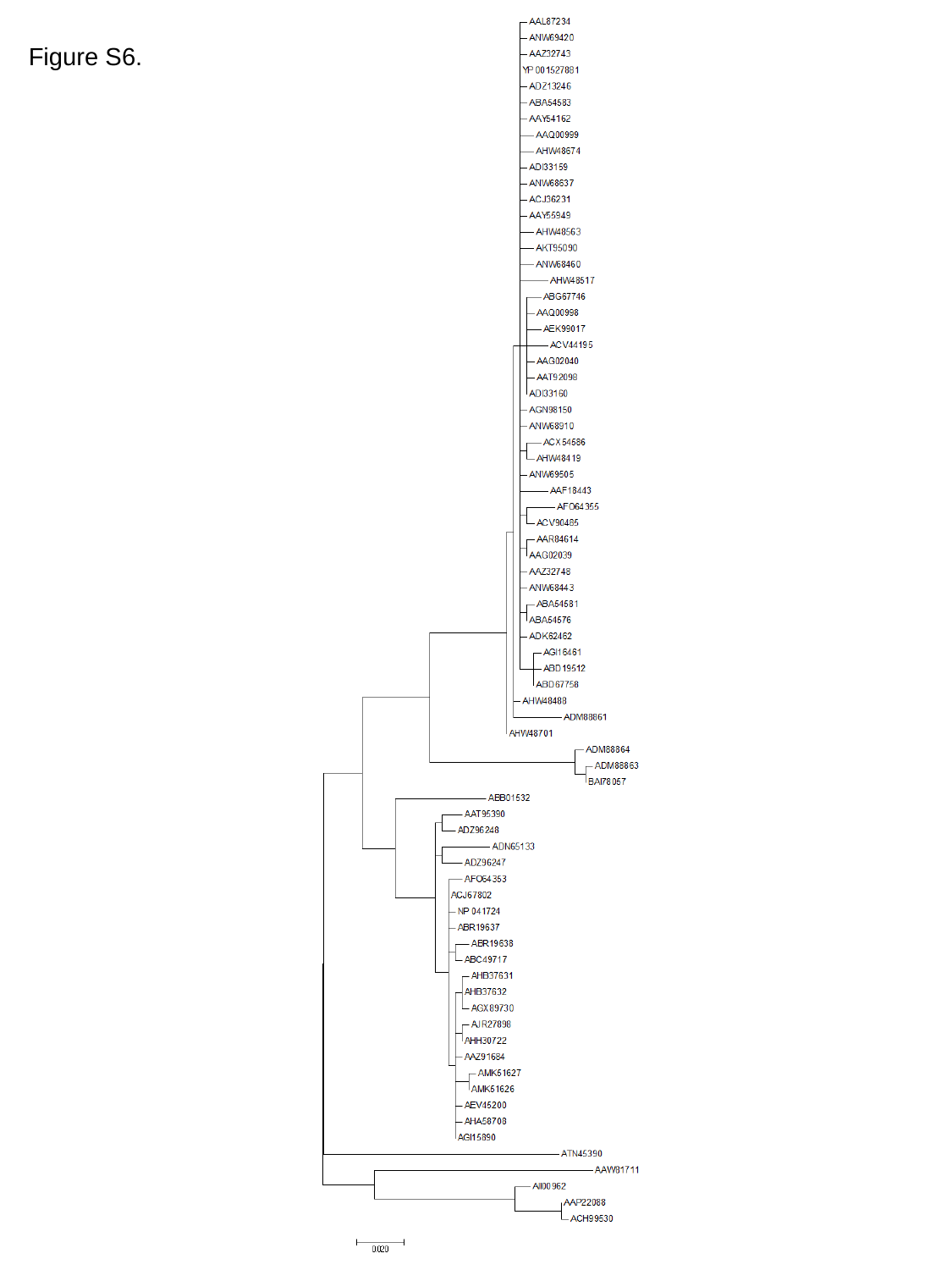

Figure S6.

## Slide 7
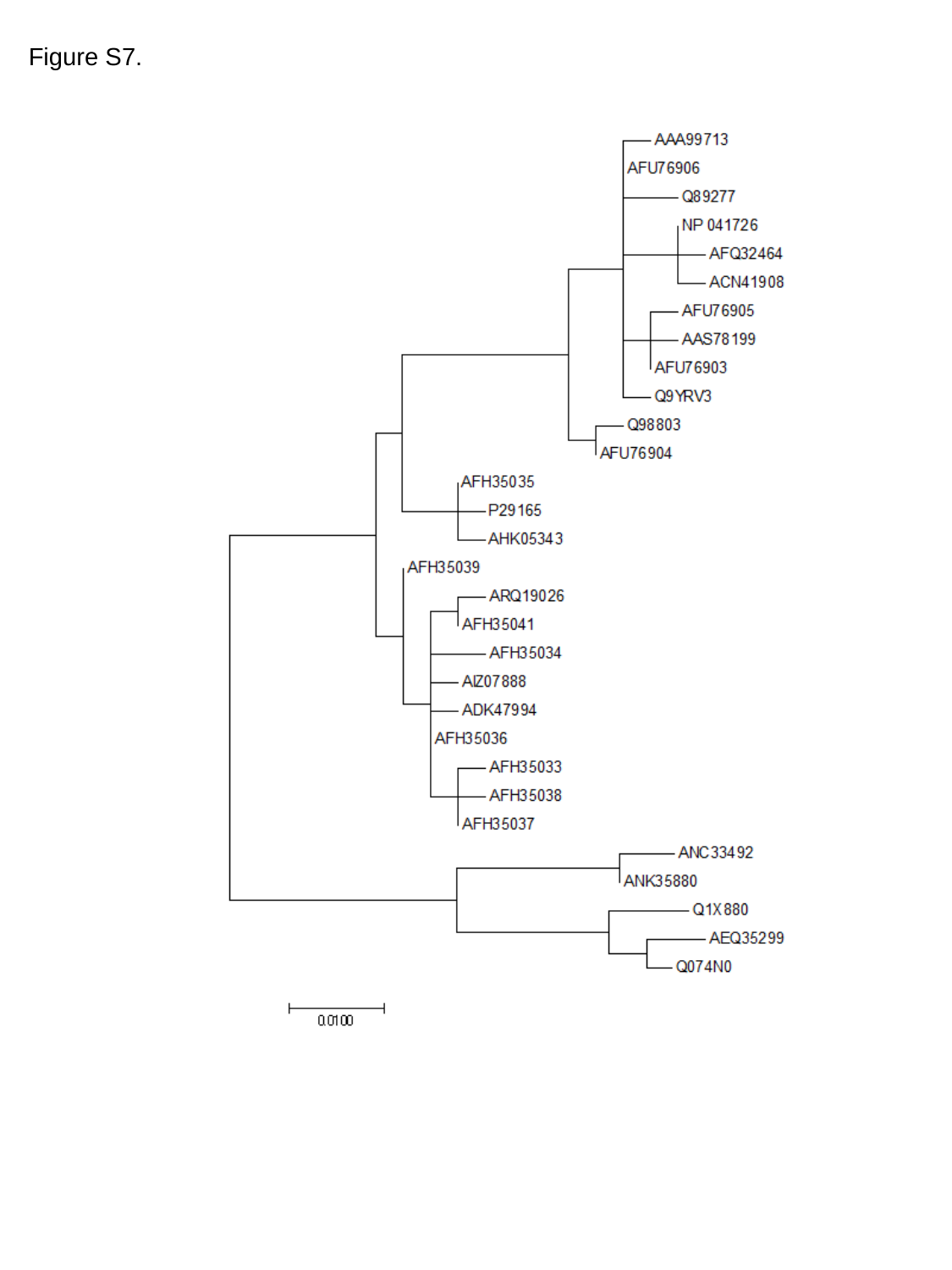

Figure S7.

## Slide 8
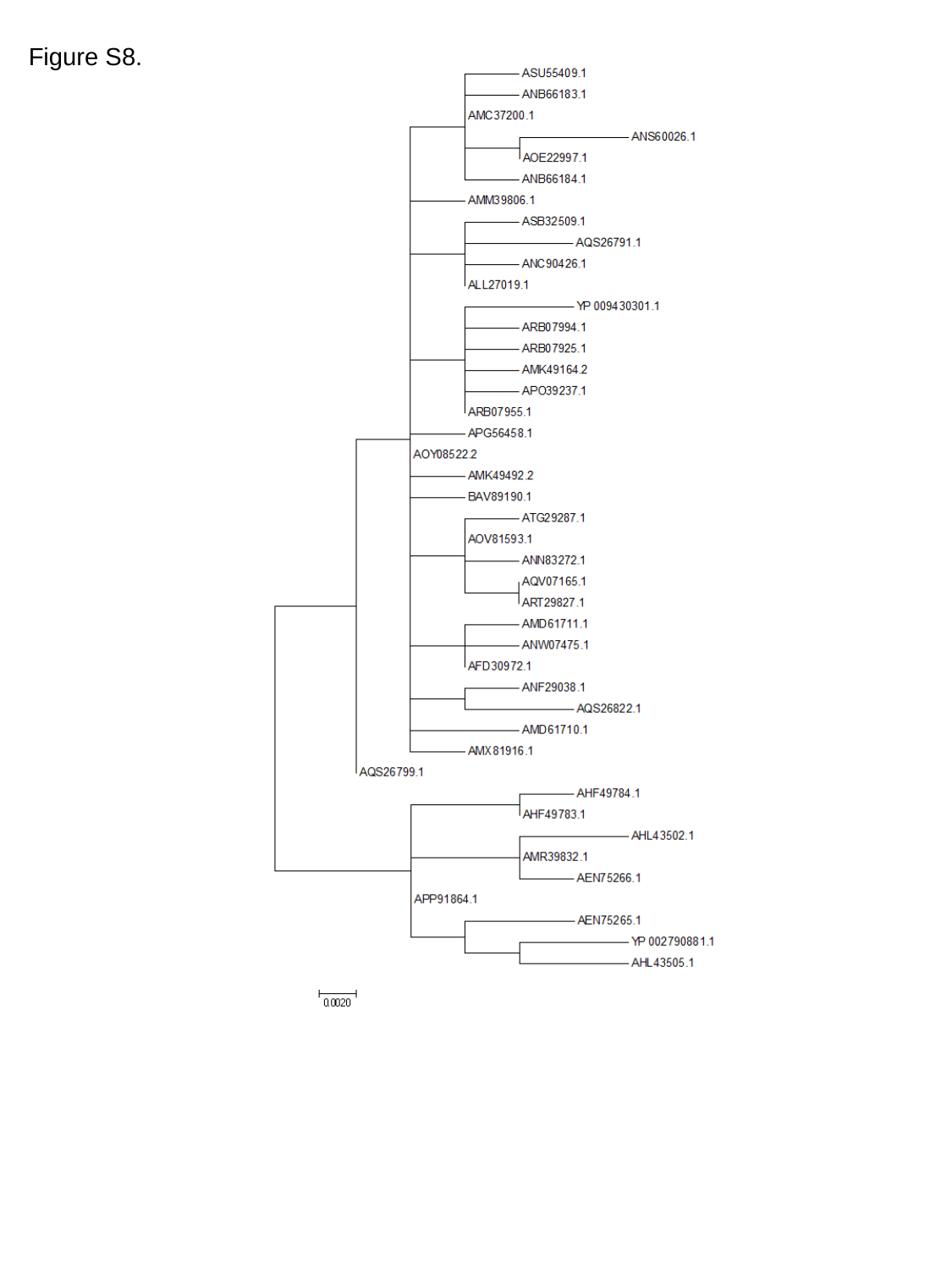

Figure S8.
